# Trichinæ in Man

**Published:** 1881-05

**Authors:** 


### TRICHINÆ IN MAN.

It has been previously stated, that for some thirty years subsequent to the first description of the capsule by Hilton, and some twenty-five years after the identification of the parasite itself in man, the same were looked upon as mere harmless curiosities, and, that, although Leidy discovered the parasite in the flesh of swine in 1847, still it was not until 1860 that the connection was established between them, appearing, as they had, in two totally different species (men and swine). The honor of this important discovery belongs to Dr. Zenker, of Dresden, Germany. The disease discovered in a servant-girl admitted as a typhus patient to the City Hospital in Dresden.

She died, and her flesh was found to be completely infested with trichinæ.

Leuckart's and other experiments have shown that a temperature of 140° F. is necessary to securely render trichinæ inert. Direct heat applied to the slides holding specimens of trichinæ pork, by means of the Shultz heating-table, has demonstrated, under the microscope, that a temperature of 50° C. (122° F.) is necessary to the certain death of the trichinæ.

Leisering's experiments with trichinous pork, made up into sausage-meat and cooked twenty minutes, gave positive results when fed to one rabbit, and negative by another. He sums up his experiments as follows :

1. Trichinæ are killed by long-continued salting of infected meat, and also by subjecting the same for twenty-four hours to the action of smoke in a heated chamber.

2. They are not killed by means of *cold* smoking for a period of three days, and it also appears that twenty minutes cooking freshly prepared sausage-meat is sufficient to kill them in all cases.

The various kinds of cooking, however, are quite different in their effects on trichinous pork. Frying and broiling are most efficient, roasting coming next. Boiling coagulates the albumen on the outer surface, and allows the heat to penetrate less readily ; it should be kept up, therefore, for at least two hours for large pieces of meat. Whether boiled, broiled or fried, pork should always be thorougly cooked.

Practically speaking, the cooking, salting and hot smoking which pork in its various forms receives in the United States, must be in the vast majority of cases sufficient to kill the trichinæ and prevent infection of the persons consuming the meat. Epidemics like those reported in Germany are unknown here, and trichiniasis in a fatal form is undoubtedly a rare disease. In the vicinity of the great pork packing establishments near Boston, the "spare-ribs," containing the intercostal muscles, are very largely bought and eaten by the people near by ; and trichiniasis among them has not in a single case been reported, so far as I have been able to learn. The *cuts* being thin and well cooked, any trichinæ in them are quite certain to be killed. Even when trichinæ are introduced into the intestinal canals too, they are sometimes expelled by diarrhœa, and the invasion of the system by a small number does no harm.—*American Microscopical Journal.*

